# Thresholdless coherence in a superradiant laser

**DOI:** 10.1038/s41377-024-01591-2

**Published:** 2024-09-05

**Authors:** Seung-Hoon Oh, Jinuk Kim, Junseo Ha, Gibeom Son, Kyungwon An

**Affiliations:** 1 Department of Physics and Astronomy & Institute of Applied Physics, Seoul National University, Seoul, Korea; 2 Korea Research Institute of Standards and Science, Daejeon, Korea

**Keywords:** Quantum optics, Lasers, LEDs and light sources

## Abstract

Lasing threshold in the conventional lasers is the minimum input power required to initiate laser oscillation. It has been widely accepted that the conventional laser threshold occurring around a unity intracavity photon number can be eliminated in the input-output curve by making the so-called *β* parameter approach unity. The recent experiments, however, have revealed that even in this case the photon statistics still undergo a transition from coherent to thermal statistics when the intracavity mean photon number is decreased below unity. Since the coherent output is only available above the diminished threshold, the long-sought promise of thresholdless lasers to produce always coherent light has become questionable. Here, we present an always-coherent thresholdless laser based on superradiance by two-level atoms in a quantum superposition state with the same phase traversing a high-Q cavity. Superradiant lasing was observed without the conventional lasing threshold around the unity photon number and the photon statistics remained near coherent even below it. The coherence was improved by reducing the coupling constant as well as the excited-state amplitude in the superposition state. Our results pave a way toward always-coherent thresholdless lasers with more practical media such as quantum dots, nitrogen-vacancy centers and doped ions in crystals.

## Abstract (web summary)

Coherent photon statistics is demonstrated regardless of the mean photon number in a high-Q thresholdless superradiant laser by phase-aligned two-level atoms.

## Graphical abstract

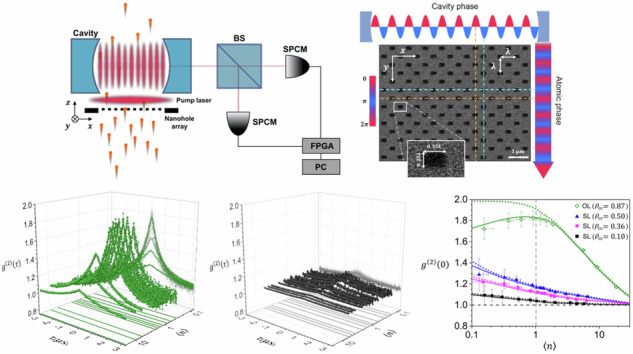

## Introduction

The conventional laser necessitates a specific amount of input power, known as the lasing threshold^1–3^, in order to initiate laser oscillation. In quantum optics, the lasing threshold is regarded as a transition point where the intracavity mean photon number reaches an order of unity so as to make the stimulated emission process become stronger than that of spontaneous emission beyond the threshold^4^. The transition from a non-lasing state to a lasing one also shares similarities with the second-order phase transition observed in condensed matter systems^5–7^. The existence of a lasing threshold is often attributed to the energy loss to non-lasing modes. Numerous efforts have been made to minimize such loss and preserve energy. In the previous theoretical studies, it has been suggested to increase the energy transfer efficiency to a specific lasing mode from total emission, in order to realize threshold-free or thresholdless lasers. This efficiency is often formulated by the so-called *β* parameter^8–10^. Lowering the lasing threshold has been demonstrated by reducing the cavity mode volume down to or even below the wavelength scale, thereby greatly enhancing the Purcell effect^11–15^. Micro- and nanoscale-cavities have been demonstrated to exhibit *β* approaching unity by reducing the number of cavity modes coupled to the gain medium^16,17^.

However, it has recently been pointed out that high *β* alone does not guarantee the coherency of the output field for small intracavity mean photon numbers^18–20^. The recent experiments have reported thermal photon statistics below the conventional threshold although they observed a smooth output-vs.-input curve in the log-log scale without exhibiting any abrupt change near the threshold. This observation strongly suggests that the conventional threshold may persist as a phase transition point in the photon statistics even when the input-output curve does not indicate the existence of the threshold.

It is noteworthy that the semiclassical theory employing the Fokker-Planck equation for the interaction between coherently pumped atoms and a cavity field predicts that these atoms emit coherent photons for any input power levels^21,22^. In the so-called superradiant laser, a strong output could be generated with the mean photon number much less than unity in the bad cavity limit due to superradiance when a large number of trapped atoms in the cavity were pumped repeatedly in pulses^23–26^. Recently, the continuous-wave single-atom superradiance by utilizing the phase correlation among *N* coherently prepared atoms traversing a high-Q cavity one by one has been achieved by employing a phase mask called a nanohole array, exhibiting *N*^2^ scaling in the mean photon number^27^.

In this paper, we report a thresholdless superradiant laser producing coherent light regardless of the intracavity mean photon numbers. In our experiments, we investigated the threshold behavior and the photon statistics of a superradiant laser in the setting of the single-atom superradiance. The conventional lasing threshold associated with the *β* parameter did not exist in the output-vs.-input curve when phase-correlated atoms (i.e., atoms prepared in a coherent superposition state) were used as a gain medium. We measured the second-order correlation of the output field in a Hanbury-Brown-Twiss configuration^28^ for various intracavity mean photon numbers selected between 0.1 and 10, with the range well enclosing the expected location of the conventional lasing threshold. We observed the coherent photon statistics persist even when the mean photon number was much less than unity. The coherence of the output was improved by reducing the excited-state population in the atomic superposition state and also by decreasing the atom-field coupling constant *g*. Our results, with a large dynamic range of superradiant lasing achievable by choosing small *β* parameter, suggest a possible way to achieve always-coherent thresholdless superradiant lasing with a practical medium such as semiconductor quantum dots^29^, nitrogen-vacancy centers^30,31^ and doped ions in crystals^32^.

## Results

The conventional superradiance requires a large number of excited atoms in a confined volume in order to be initiated^33,34^. Recently, superradiance has been realized even when a high-Q cavity contains fewer atoms than one on average^27^. A phase mask for atoms, called a nanohole array^35^, was used to enforce phase correlation among atoms in the superposition state pumping as well as in their transit through the cavity during the interaction time *τ*. As a result, the atoms are initially prepared in a superradiant state described by^27^

1 $${\left|\Psi \right\rangle }_{{\rm{a}}}=\mathop{\prod }\limits_{i=1}^{N}[\cos (\Theta /2){\rm{|}}{\text{g}}{{{\rangle }}}_{i}+{e}^{i{\phi }_{0}}\sin (\Theta /2){\rm{|}}{\text{e}}{{{\rangle }}}_{i}]$$

where *N* is the number of atoms interacting with the cavity, *i* denotes the atom index, g(e) denotes the ground(excited) state of the atom, *ϕ*_0_ is the common relative phase shared by all atoms and Θ is the pump pulse area or *π* − Θ is the azimuthal angle in the *N*-atom Bloch sphere. The superradiant state given by Eq. (1) can undergo superradiance immediately without the usual time delay accompanied in the conventional superradiance. In addition, the cavity helps to preserve the phase correlations among successive atoms during the cavity-field decay time $1/{\gamma }_{{\rm{c}}}$. As a result one atom on average in the cavity ($\bar{N}=1$) corresponds to ${N}_{{\rm{c}}}=\bar{N}/({\gamma }_{{\rm{c}}}\tau )\gg 1$ atoms interacting with the cavity mode effectively with ${\gamma }_{{\rm{c}}}\tau \ll 1$, so superradiance can still occur with $\bar{N} < 1$.

The average mean photon number $\langle{{n}}\rangle$ and the photon statistics inside the cavity in the steady state can be obtained by solving the quantum master equation with the Jaynes-Cummings Hamiltonian extended to multiple atoms as shown in Supplementary Note 1. The result is

2 $${{\langle }}n{{\rangle }}=\frac{{\gamma }_{{\rm{c}}}}{{\Gamma }_{{\rm{c}}}^{{\prime} }}{\rho }_{{\rm{ee}}}{(g\tau )}^{2}{N}_{{\rm{c}}}+{\left[\frac{2{\gamma }_{{\rm{c}}}}{{\Gamma }_{{\rm{c}}}^{{\prime} }}|{\rho }_{{\rm{eg}}}|\left(g\tau \right){N}_{{\rm{c}}}\right]}^{2}$$

where ${\Gamma }_{{\rm{c}}}^{{\prime} }=2{\gamma }_{{\rm{c}}}\left[1+\left(\frac{1}{2}-{\rho }_{{\rm{ee}}}\right){\left(g\tau \right)}^{2}{N}_{{\rm{c}}}\right]$ is the modified photon decay rate due to the atom-field interaction, *g* is the atom-cavity coupling constant, ${\rho }_{{\rm{ee}}}={\sin }^{2}(\Theta /2)$ is the atomic-density-matrix diagonal element corresponding to the excited-state population, and ${\rm{|}}{\rho }_{{\rm{eg}}}{\rm{|}}=\sin (\Theta /2)\cos (\Theta /2)$ is the atomic-density matrix off-diagonal element corresponding to the atomic coherence. The first term, originating from the atomic population, corresponds to the non-collective emission. Below the conventional lasing threshold, it equals the number of thermal photon, ${n}_{{\rm{th}}}$, in the cavity. On the other hand, the second term occurs only when the atomic phase correlation exists. The quadratic dependency on the atom number *N*_c_ indicates it corresponds to superradiance with the mean photon number denoted as *n*_sr_. Its photon number distribution is given by a Poisson distribution ${P}_{{\rm{sr}}}\left(n\right)\propto \tfrac{{\left({\left|\alpha \right|}^{2}\right)}^{n}}{n!}$, corresponding to a coherent state ${{|}}\alpha {{\rangle }}$ with ${\rm{\alpha }}=-i\left(2{\gamma }_{{\rm{c}}}/{\Gamma }_{{\rm{c}}}^{{\prime} }\right){N}_{{\rm{c}}}(g\tau ){\rho }_{{\rm{eg}}}$^27^ (see Supplementary Note 1 for derivation).

The present superradiant lasing in a high-Q cavity does not exhibit the conventional threshold. This can be understood as follows. When ${N}_{{\rm{c}}}\ll 1$, each atom undergoes spontaneous emission independently, and only a small fraction corresponding to $\beta \approx {\left(g\tau \right)}^{2}$ is captured by the cavity. On the other hand, when ${N}_{{\rm{c}}}\gg 1$, individual atoms are phase correlated with each other due to the enforced phase matching condition by the nanohole array. The emission from atoms can constructively interfere in the direction of the cavity mode, resulting in the superradiance. As *N*_c_ is increased beyond unity, because of ${N}_{{\rm{c}}}^{2}$ scaling of the superradiance, the ratio of the collective- to the non-collective emission monotonically increases as *N*_c_. As a result, there exists no additional transition like the conventional threshold once superradiance starts around ${N}_{{\rm{c}}}=1$.

In the experiment, $g\tau \ll 1$ is satisfied, corresponding to the Markovian regime. Under this condition, we have ${\Gamma }_{{\rm{c}}}^{{\prime} }\simeq 2{\gamma }_{{\rm{c}}}$, and thus at the superradiance threshold ${N}_{{\rm{c}}}=1$, $\left\langle n\right\rangle \simeq {\left(g\tau \right)}^{2}{\sin }^{2}\left(\frac{\Theta }{2}\right)\left\{\frac{1}{2}+{\cos }^{2}\left(\frac{\Theta }{2}\right)\right\} \sim {\left(g\tau \right)}^{2}\ll 1$. Therefore, one can choose the value of *gτ* as small as possible in order to lower the smallest mean photon number the superradiance can generate. Consequently, reducing *gτ* has an advantage of observing the more coherent field for a target mean photon number.

Note that the ${N}_{{\rm{c}}}^{2}$-growth of the mean photon number is eventually saturated due to the coherent Rabi oscillation. In the semiclassical limit of ${{\langle }}n{{\rangle }}\gg 1$, starting from the initial azimuthal angle ($\pi -\Theta$) in the *N*-atom Bloch sphere, the state vector rotation angle $\sqrt{n}g\tau$ larger than Θ would result in reabsorption of the emitted photons or saturation. In the limit of $(g\tau )\ll 1\ll {{\langle }}n{{\rangle }}$, we have $\left\langle n\right\rangle \simeq {n}_{{\rm{sr}}}=\frac{1}{4}{\left[\sin \Theta \left(g\tau \right){N}_{{\rm{c}}}\right]}^{2}$, so in order to avoid the saturation, *N*_c_ should be kept smaller than ${N}_{{\rm{c}},{\rm{sat}}}\left(\Theta \right)=2\left(\Theta /\sin \Theta \right){\left(g\tau \right)}^{-2}$, and at ${N}_{{\rm{c}}}={N}_{{\rm{c}},{\rm{sat}}}$ we have ${\left\langle n\right\rangle }_{{\rm{sat}}}={\left[\Theta /\left(g\tau \right)\right]}^{2}\sim {\left(g\tau \right)}^{-2}$.

Since the dynamic range of the mean number of photons generated by superradiance spans from ${\left(g\tau \right)}^{2}$ to ${\left(g\tau \right)}^{-2}$, it is again preferable to have *gτ* values as small as possible. In the experiment to be discussed below, the dynamic range of the mean photon number covers about 4 orders of magnitude from about 0.01 to 100 (with ${\rho }_{{\rm{ee}}}=0.50$) and in this range coherent output is expected because of the superradiance.

**Fig. 1:**
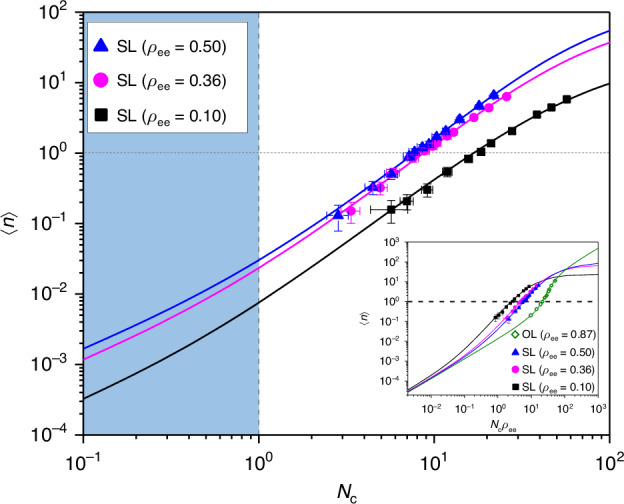
**The input-output curve of the superradiant lasing**. The intracavity mean photon number ${{\langle }}n{{\rangle }}$ proportional to the output power is plotted as a function of ${N}_{{\rm{c}}}$ proportional to the input power for three different phase-correlated atomic superposition states represented in the excited state population ${\rho }_{{\rm{ee}}}=0.10$($\Theta =0.64$, black squares), 0.36 ($\Theta =1.29$, magenta circles) and 0.50 ($\Theta =1.58$, blue triangle). The slope fits (not shown) in the log-log scale are $1.78\pm 0.13$(black), $1.84\pm 0.05$(magenta), and $1.93\pm 0.06$(blue), respectively. The superradiant threshold appears at ${N}_{{\rm{c}}}=1$, well below which (shaded region) spontaneous emission dominates and thus all three curves converge to a unity-slope curve when plotted as a function of ${\rho }_{{\rm{ee}}}{N}_{{\rm{c}}}$ as shown in the inset. The solid curves are the solutions of the master equation describing the system. The input-output curve for ${\rho }_{{\rm{ee}}}=0.87$ (olive-colored diamonds and its associated theoretical curve shown as a solid olive-colored curve) corresponds to the conventional lasing (see the main text for more information) with a conventional threshold around unity photon number. The error bars indicate standard deviations from repeated measurements

In order to verify the absence of the conventional threshold in the present superradiant lasing, we first measured the output power as a function of the input power. The former is represented by the mean photon number ${{\langle }}n{{\rangle }}$ in the cavity and the latter is formulated in terms of the mean number *N*_c_ of atoms injected into the cavity during the cavity field decay time ${\gamma }_{{\rm{c}}}^{-1}$. The result is summarized in Fig. 1. Three different superposition states were tried with ${\rho }_{{\rm{ee}}}=0.10$, 0.36 and 0.50 with 0.01 uncertainty for each. For all, we observe no slope change around $\left\langle n\right\rangle =1$ (horizontal dotted line), at which the conventional lasing threshold is expected if the present lasing were of ordinary one. Slope fits in the region $0.1 < \left\langle n\right\rangle < 10$ are 1.78 ± 0.13, 1.84 ± 0.05, and 1.93 ± 0.06, respectively, all approaching 2, and thus signifying superradiant lasing (SL).

Similar data exhibiting no slope change around unity photon number was previously obtained in the single-atom superradiance experiment^27^. The present data re-establish the absence of the conventional threshold in the superradiant lasing and set the stage for the photon statistics study to be presented below.

As discussed in the preceding section, the superradiant lasing occurs in a region specified by $1 < {N}_{{\rm{c}}} < {N}_{{\rm{c}},{\rm{sat}}}=2\left(\Theta /\sin \Theta \right){\left(g\tau \right)}^{-2}$. The predicted values ${N}_{{\rm{c}},{\rm{sat}}}$ for the three cases in Fig. 1 are 84, 105, 123 for ${\rho }_{{\rm{ee}}}=0.10,0.36$ and 0.50, respectively, and the range of the corresponding mean photon numbers in the superradiant lasing are $0.0035\sim 16,0.011\sim 65,0.013\sim 98$, respectively. These predicted values are in a fair agreement with the observation in Fig. 1.

The input-output curves are replotted in the inset of Fig. 1 as a function of ${\rho }_{{ee}}{N}_{{\rm{c}}}$, parameterizing the excited-state contribution in *N*_c_. Well below the superradiance threshold ${N}_{{\rm{c}}}=1$, the atomic emission is characterized by spontaneous emission, which is proportional to the excited state population. Therefore, all three curves should converge to a common line of slope of unity, which is confirmed in the inset. We also plot the case of ${\rho }_{{\rm{ee}}}=0.87\pm 0.01$ (green diamonds), for which atoms are prepared without phase correlation (see Methods), leading to an ordinary lasing (OL) with a clear lasing threshold indicated by an abrupt change in the slope in the log-log plot. We note the center of lasing threshold around ${N}_{{\rm{c}}} \sim 30$ with ${{\langle }}n{{\rangle }}\sim 2$, confirming our expectation of the conventional threshold to appear around unity photon number. This is a clear contrast to the absence of such a threshold in the superradiant lasing.

**Fig. 2:**
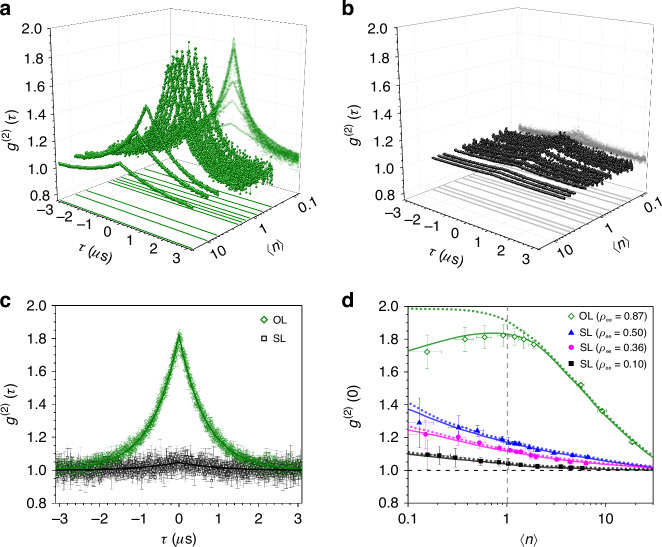
The second-order correlation ${{\boldsymbol{g}}}^{{\boldsymbol{(}}{\bf{2}}{\boldsymbol{)}}}{\boldsymbol{(}}{\boldsymbol{t}}{\boldsymbol{)}}$ of the superradiant lasing. **a** Evolution of ${g}^{(2)}(t)$ of the ordinary lasing (OL) employing excited atoms with ${\rho }_{{\rm{ee}}}=0.87(\Theta =2.63)$ without atomic phase correlation for various ${{\langle }}n{{\rangle }}$ values sampled between 0.1 and 10. **b** The same for the superradiant lasing (SL) using the superposition state with ${\rho }_{{\rm{ee}}}=0.10$ ($\Theta =0.64$). **c** Comparison of the two cases with $\Theta =\pi$ (OL, in olive-colored circles) and $\Theta =0.64$ (SL, in black circles) for the same mean photon number $\left\langle n\right\rangle =1.0$, near the conventional threshold of the ordinary lasing. **d** The second-order correlation at zero time delay, ${g}^{(2)}(0)$, as a function of the intracavity mean photon number ${{\langle }}n{{\rangle }}$ for ${\rho }_{{\rm{ee}}}=0.87$ (OL) and ${\rho }_{{\rm{ee}}}=\mathrm{0.10,0.36,0.50}$ (SL), respectively. The solid curves in (**a**–**c**) are fits given by Eq. (S21) in Supplementary Note 1. In (**d**), solid(dotted) curves are polynomial fits of the quantum trajectory simulation results including(excluding) the effect of background counts. The error bars indicate standard deviations from repeated measurements

Next, in order to verify the coherent photon statistics over the range of the mean photon numbers corresponding to the superradiant lasing, we measured the second-order correlation ${g}^{(2)}(t)$ of the output photons employing the Hanbury-Brown-Twiss method (see Methods for experimental details). The results are summarized in Fig. 2.

Firstly, the second-order correlation shown in Fig. 2a is the one obtained with ${\rho }_{{\rm{ee}}}=0.87$ without using the nanohole array as a comparison set. In this case, an ordinary lasing takes place. Around the unity photon number, we note photon statistics undergoes a transition from coherence (for ${{\langle }}n{{\rangle }}\gg 1$) to thermal statistics (for ${{\langle }}n{{\rangle }}\ll 1$). This transition is more clearly seen in Fig. 2d plotting the second-order correlation at zero delay time, ${g}^{(2)}(0)$.

Figure 2b summarizes the evolution of ${g}^{(2)}(t)$ for superradiant lasing with ${\rho }_{{\rm{ee}}}=0.10$($\Theta =0.64$) as the mean photon number changes from 0.1 to 10. The evolution of the second-order correlation at zero delay time ${g}^{(2)}(0)$ is presented in Fig. 2d for three different ${\rho }_{{\rm{ee}}}$ values, 0.10, 0.36, and 0.50. Across the board, ${g}^{(2)}(0)$ remains close to unity, indicating the photon statistics are close to those of coherent light and particularly there is no abrupt change of ${g}^{(2)}(0)$ around unity photon number, in striking contrast to the case of ordinary lasing. As ${{\langle }}n{{\rangle }}$ is decreased, the deviation of ${g}^{(2)}(0)$ from 1 gradually grows although the deviation is much less than 1. This gradual increase is due to the contribution from non-collective emission of thermal photon statistics, corresponding to the first term in Eq. (2). We can note that the value of [${g}^{\left(2\right)}\left(0\right)-1$] is reduced as ${\rho }_{{\rm{ee}}}$ is decreased because of the reduction of spontaneous emission. For ${\rho }_{{\rm{ee}}}=0.10$, even in the region of thermal photon statistics for the case of OL such as $\left\langle n\right\rangle \sim 0.1$, we have ${g}^{\left(2\right)}\left(0\right)\simeq 1.1$, remaining quite coherent. The correlation time of ${g}^{\left(2\right)}\left(t\right)$ for the superradiant lasing in Fig. 2b, c is given by $\frac{1}{{\Gamma }_{{\rm{c}}}^{{\prime} }}=\frac{1}{2{\gamma }_{{\rm{c}}}}{[1+0.5\cos \Theta {(g\tau )}^{2}{N}_{{\rm{c}}}]}^{-1}\approx \frac{1}{2{\gamma }_{{\rm{c}}}}$. The correlation time for the ordinary lasing in Fig. 2c is larger than $\frac{1}{2{\gamma }_{{\rm{c}}}}$ due to the increased fluctuation near the conventional threshold^36^.

## Discussion

The increase of $\left[{g}^{\left(2\right)}\left(0\right)-1\right]$ as $\left\langle n\right\rangle$ is decreased as shown in Fig. 2 can be quantified by introducing a measure *M* of the dominance of collective contribution as $M={n}_{{\rm{sr}}}/{n}_{{\rm{th}}}=\frac{4{\gamma }_{{\rm{c}}}}{{\Gamma }_{{\rm{c}}}^{{\prime} }}{\rho }_{{\rm{gg}}}{N}_{{\rm{c}}}$. One can show (see Supplementary Note 1)

3 $${g}^{\left(2\right)}\left(0\right)-1=\frac{1+2M}{{(1+M)}^{2}}$$

which monotonically decreases from 1 to 0 as *M* is increased: for $M\ll 1$ we get ${g}^{\left(2\right)}\left(0\right)\approx 2$ whereas for $M\gg 1$ we obtain ${g}^{\left(2\right)}\left(0\right)\approx 1$. For a given choice of ${\rho }_{{\rm{ee}}}$ (= $1-{\rho }_{{\rm{gg}}}$), as we reduce ${N}_{{\rm{c}}}$, 〈$n$〉 as well as *M* are decreased to make ${g}^{\left(2\right)}\left(0\right)$ go up as in Fig. 2d.

**Fig. 3:**
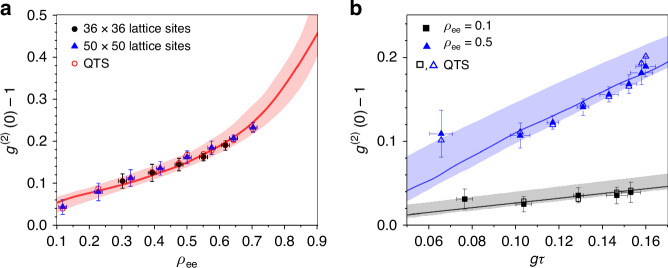
Population and ${\boldsymbol{g}}{\boldsymbol{\tau }}$ dependence of the second-order correlation near the conventional threshold. **a** The second-order correlation ${g}^{(2)}(0)$ at zero delay time as a function of the excited-state population ${\rho }_{{\rm{ee}}}$ in the composition of the phase-correlated superposition state_._ The atom number ${N}_{{\rm{c}}}$ is adjusted to keep the mean photon number ${{\langle }}n{{\rangle }}\simeq 1$ around the conventional lasing threshold. Two different nanohole arrays were employed. **b** The same, but ${\rho }_{{\rm{ee}}}$ is fixed at 0.10 or 0.50 and $g\tau$ is varied from 0.065 to 0.16 while keeping the mean number of photons close to 1. The actual mean photon numbers vary between 0.92 and 1.08 among the data points in (**a**) and between 0.88 and 1.03 for ${\rho }_{{\rm{ee}}}=0.1$ and between 0.90 and 1.12 for ${\rho }_{{\rm{ee}}}=0.5$ in (**b**). The quantum trajectory simulation (QTS) results with the actual mean photon numbers are shown as open symbols for individual data points. The shades indicate the bounds set by the QTS results. The solid curves in (**a**, **b**) represent the QTS results for exactly $\left\langle n\right\rangle =1$ for guidance. The error bars indicate standard deviations from repeated measurements. The mechanical vibrations and thermal drift of the nanohole array as well as the oven temperature change during the measurement time contribute mostly to the variances associated with the vertical and horizontal error bars. The large error bars for small $g\tau$ is due to limited measurement time because of clogging the nanoholes at high atomic beam flux to produce the same mean photon number (see “Methods” section for details)

We are interested in the photon statistics when $\left\langle n\right\rangle \simeq 1$, around which the conventional threshold takes place for OL, exhibiting thermal photon statistics. Figure 3a shows the observed ${g}^{\left(2\right)}\left(0\right)$ for superradiant lasing as a function of ${\rho }_{{\rm{ee}}}$ under the condition $\left\langle n\right\rangle =1$. For different ${\rho }_{{\rm{ee}}}$, ${N}_{{\rm{c}}}$ is adjusted to keep the mean photon number at unity.

Under the present experimental conditions, $\left\langle n\right\rangle =1$ occurs with ${N}_{{\rm{c}}}\gg 1$, so the superradiance dominates in Eq. (2). With the mean photon number fixed, ${\rho }_{{\rm{eg}}}{N}_{{\rm{c}}}$ would remain constant (under the Markovian approximation, $2{\gamma }_{{\rm{c}}}/{\Gamma }_{{\rm{c}}}^{{\prime} }\approx 1$). Since ${N}_{{\rm{c}}}\propto |{\rho }_{{\rm{eg}}}{|}^{-1}=1/\sqrt{{\rho }_{{\rm{ee}}}(1-{\rho }_{{\rm{ee}}})}$, the parameter *M* varies as $M\propto \sqrt{{\rho }_{{\rm{ee}}}^{-1}-1}$. As ${\rho }_{{\rm{ee}}}$ is decreased, therefore, *M* gets larger and thus ${g}^{\left(2\right)}\left(0\right)$ becomes smaller.

**Fig. 4:**
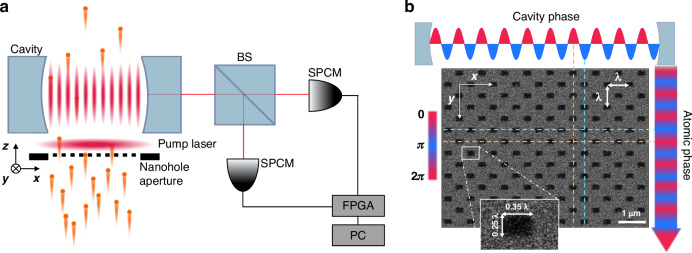
Experimental setup. **a** The experimental schematic for photon statistic measurement. ^138^Ba atoms in a beam go through a nanohole array with the hole spacing equal to the atomic transition wavelength and are exposed to a pump field (in ${{y}}$ direction) to be excited to a superposition state with the same phase for all atoms. The nanoholes are aligned with the antinodes of a high-Q cavity resonant with the atom. The output from the cavity is fed to the Hanbury-Brown-Twiss arrangement made of a 50:50 beam-splitter (BS) and two single-photon counting modules (SPCMs). The second-order correlation of the output field is obtained from all possible pairs of counting events on two detectors recorded with a field programmable gate array (FPGA). **b** The scanning electron microscope image of the nanohole array used in the superradiant lasing experiment. Each rectangular hole measures $0.35\lambda \times 0.25\lambda$

In Fig. 3b, ${g}^{\left(2\right)}\left(0\right)$ is measured as a function of *gτ* for a fixed $\left\langle n\right\rangle$ value of unity. In experiments, *τ* determined by the velocity of atoms in a beam is fixed and *g* is varied by adjusting the vertical location [in *y* in Fig. 4b] of the nanohole array with respect to the cavity mode (along *x*) (see Methods for details). Note that the parameter *M* does not have explicit dependence on *gτ*. The dependence comes through *N*_c_ for keeping the mean photon number constant for different *gτ* values. Again, from Eq. (2), $|{\rho }_{{\rm{eg}}}|g\tau {N}_{{\rm{c}}}=$ constant, and thus $M\propto \left(1-{\rho }_{{\rm{ee}}}\right){N}_{{\rm{c}}}\propto \frac{1}{g\tau }\sqrt{{\rho }_{{\rm{ee}}}^{-1}-1}$, which explains why $\left[{g}^{\left(2\right)}\left(0\right)-1\right]$ gets smaller as *gτ* is reduced. More intuitively, for smaller *gτ*, we need larger *N*_c_ to keep the same photon number and this makes *M* larger and thus $\left[{g}^{\left(2\right)}\left(0\right)-1\right]$ smaller.

Our results in Figs. 1–3 indicate that it is better to make *gτ* as small as possible in order to extend the dynamic range, spanning from ${\left(g\tau \right)}^{2}$ to ${\left(g\tau \right)}^{-2}$, of superradiant lasing as much as possible. Moreover, it is advantageous to employ ${\rho }_{{\rm{ee}}}$ as small as possible in the phase-correlated atomic superposition state given by Eq. (1) in order to make *M* as large as possible and thus to make $\left[{g}^{\left(2\right)}\left(0\right)-1\right]$ as small as possible, *i.e*., more coherent output. Smaller *gτ*, in addition to the advantage of larger dynamic range, also helps to make more coherent output for a given mean number of photons in the cavity.

In conclusion, we have investigated the photon statistics of a superradiant laser operating with two-level atoms prepared in the same superposition state traversing a high-Q cavity. Superradiance was observed when *N*_c_, the number of atoms traversing the cavity during the cavity-field decay time, is larger than unity. Although the so-called *β* parameter was much less than unity, once the superradiance occurs with ${N}_{{\rm{c}}} > 1$, the mean intracavity photon number versus the mean intracavity atom number in the log-log plot did not exhibit the conventional lasing threshold, which is expected around a unity mean photon number. The photon statistics obtained from the measured second-order correlation function remained close to coherent statistics, not exhibiting any threshold behavior across the unity photon number. It was in contrast to the clear transition between thermal and coherent photon statistics observed in the ordinary lasing performed in the same setup with population inversion only. The coherence of the output was improved by reducing the excited-state population in the atomic superposition state and also by decreasing the atom-field coupling constant *g*. The dynamic range of superradiant lasing spanned four orders of magnitude in the intracavity mean photon number. Our result suggests a possible route to take for realizing always-coherent thresholdless lasers independent of the intracavity photon number based on practical gain media such as quantum dots, nitrogen-vacancy centers, and doped ions in crystals.

## Materials and methods

The schematic of our experimental setup is shown in Fig. 4a. ^138^Ba atoms in a beam traverse a high-Q Fabry-Perot cavity in the *z* direction. We utilize ^1^S_0_ and ^3^P_1_ levels as two levels interacting with the cavity resonance. The atoms in the ground state go through a nanohole array in Fig. 4b with hole spacing in both *x* and *y* directions matched with the transition wavelength of the ^1^S_0_ ↔ ^3^P_1_ transition ($\lambda =791{\rm{nm}}$) before they are excited to a superposition state by a traveling-wave pump laser field propagating in the *y* direction. The nanoholes are aligned with the antinodes of the TEM_00_ mode of the cavity. As a result, the atoms are excited to the superposition state as in Eq. (1) with a common relative phase *ϕ*_0_ for all atoms. The pump intensity is adjusted to achieve a desired pulse area Θ. For the superradiant lasing experiment, the strong coupling condition is satisfied with $(\bar{g} ,{\gamma }_{{\rm{a}}},{\gamma }_{{\rm{c}}})/2\pi =(256,25,119){\rm{kHz}}$, where $\bar{g}$ represents the atom-cavity coupling spatially averaged over individual nanoholes and ${\gamma }_{{\rm{a}}}$(${\gamma }_{{\rm{c}}}$) corresponds to the atomic (cavity) decay rate (both half widths).

For the ordinary lasing experiment, the nanohole array was replaced with a rectangular aperture (250 µm × 25 µm) and the atomic beam was tilted by 0.22 mrad in order to achieve the traveling-wave atom-cavity interaction^36^. Atoms were fully excited with the pump pulse area Θ close to *π*. The actual excited state population was ${\rho }_{{\rm{ee}}}=0.87\pm 0.01$ because of the finite width $\Delta v\simeq 0.24{v}_{0}$ of the velocity distribution of the atomic beam with the mean velocity ${v}_{0}\simeq 820\,{\rm{m}}{{\rm{s}}}^{-1}$. In this case, the atoms underwent non-collective emission in the cavity. The strong coupling condition was also satisfied with a reduced coupling constant $\bar{g}'/2\pi =190\,{\rm{kHz}}$ due to the traveling-wave interaction. Both experiments (SL, OL) were done in the Markovian regime ($g\tau \ll 1$) with short interaction time $\tau \simeq 0.10\,\mu {\rm{s}}$.

The photon statistics were obtained by measuring the second-order correlation of the output employing the Hanbury-Brown-Twiss arrangement composed of a 50:50 beam-splitter and two photon-counting detectors as shown in Fig. 4. These detectors, denoted as single-photon counting module had 60% quantum efficiency and low dark counts, the rate of which was less than 500 counts per second (cps). The background counts were about $8\times {10}^{3}{\rm{cps}}$ due to the scatter of the cavity locking laser beam and the thermal radiation from the atomic beam oven. The counting rate of the lasing output associated with one intracavity photon on average was about $1.0\times {10}^{5}{\rm{cps}}$ in comparison. The arrival times of photons on each detector were recorded with a field-programmable gate array and the second-order correlation was calculated in real time from all possible parings of the recorded count events^36^. The unit time or time bin for constructing the second-order correlation function was 6.3 ns.

The nanohole array aperture is prone to hole clogging due to barium atoms sticking around the holes at high atomic beam flux. For the data taken with $g\tau \,<\, 0.08$ in Fig. 3b, we had to use high atomic beam flux in order to keep the intracavity mean photon fixed number around unity. To avoid the hole clogging problem, the measurement time was set at 3 min, during which the total number of photon counting events was about 1/4 of those for other data points with $g\tau \,>\, 0.1$, thus resulting in a relatively low signal-to-noise ratio.

## Supplementary information

Supplementary Information for “Thresholdless coherence in a superradiant laser”
