# Supplementary material for: Thresholdless coherence in a superradiant laser
Source: Light Sci Appl. 2024 Sep 5;13:239. doi: 10.1038/s41377-024-01591-2 (PMC11377561; doi:10.1038/s41377-024-01591-2)
Supplement: Supplementary file 1 — Supplementary Information for “Thresholdless coherence in a superradiant laser” [file 41377_2024_1591_MOESM1_ESM.pdf]

Supplementary Information for
"Thresholdless coherence in a superradiant laser"

Seung-hoon Oh,[1] Jinuk Kim,[2] Junseo Ha,[1] Gibeom Son,[1] and Kyungwon An[1,*]

[1]*Department of Physics and Astronomy & Institute of Applied Physics, Seoul National University, Seoul 08826, Korea*

[2]*Korea Research Institute of Standards and Science, Daejeon 34113, Korea*

1. MASTER EQUATION DESCRIPTION OF SUPERRADIANT LASING

The atom-field interaction in the superradiant lasing in our experiment can be described in the interaction picture by the Tavis-Cummings Hamiltonian,

$$H_{\mathrm{AF}} = \sum_k^N H_k = \hbar g \sum_k^N \left(\sigma_k^\dagger a e^{-i\Delta t} + a^\dagger \sigma_k e^{i\Delta t} \right), \tag{S1}$$

where H_k is the Jaynes-Cummings Hamiltonian for the kth atom, g is the atom-cavity coupling constant, $\sigma_k(\sigma_k^\dagger)$ is the atomic lowering(raising) operator for the kth atom, $a(a^\dagger)$ is the photonic annihilation(creation) operator and $\Delta = \omega_0 - \omega$ represents the atom-cavity detuning with $\omega(\omega_0)$ the cavity resonance(atomic transition) frequency. Using the N-term-transposed Zassenhaus formula

$$e^{\lambda(X_1+X_2+\cdots+X_N)} = e^{\mathcal{O}\left(\lambda^3\right)} e^{\frac{1}{2}\lambda^2 \sum_{i=1}^{N-1}\sum_{j=i+1}^{N}[X_i,X_j]} e^{\lambda X_N}\cdots e^{\lambda X_1}, \tag{S2}$$

the time-evolution operator $U_{1+2+\cdots+N} = e^{-iH_{\mathrm{AF}}t/\hbar}$ of the Tavis-Cummings Hamiltonian can be expressed as a multiplication of the time-evolution operator $U_k = e^{-iH_k t/\hbar}$ of the kth atom described by the Jaynes-Cummings Hamiltonian. Under the assumption of $g\tau \ll 1$, the time evolution operator for the time interval τ can be approximated as

$$\begin{aligned} &U_{1+2+\cdots+N} \\ &= e^{\mathcal{O}((g\tau)^3)} e^{-\frac{1}{2}(g\tau)^2 \sum_{i=1}^{N-1}\sum_{j=i+1}^{N}\left(\sigma_i^\dagger\sigma_j - \sigma_i\sigma_j^\dagger\right)} U_N\cdots U_2 U_1. \end{aligned} \tag{S3}$$

To get the field density matrix after τ, we take a trace over atoms:

$$\begin{aligned} \rho^{\mathrm{F}}(\tau) &= \mathrm{Tr_a}\left[\rho^{\mathrm{AF}}(\tau)\right] \\ &= \mathrm{Tr_a}\left[U_{1+2+\cdots+N}(\tau)\rho^{\mathrm{AF}}(0)U_{1+2+\cdots+N}(\tau)^\dagger\right] \\ &= \mathrm{Tr_a}\Big[e^{\mathcal{O}\left((g\tau)^3\right)} e^{-\frac{1}{2}(g\tau)^2\sum_{i=1}^{N-1}\sum_{j=i+1}^{N}\left(\sigma_i^\dagger\sigma_j - \sigma_i\sigma_j^\dagger\right)} \\ &\quad \times U_N\cdots U_2 U_1 \rho^{\mathrm{AF}}(0) U_1^\dagger U_2^\dagger\cdots U_N^\dagger \\ &\quad \times e^{-\frac{1}{2}(g\tau)^2\sum_{i=1}^{N-1}\sum_{j=i+1}^{N}\left(\sigma_i\sigma_j^\dagger - \sigma_i^\dagger\sigma_j\right)} e^{\mathcal{O}\left((g\tau)^3\right)}\Big], \end{aligned} \tag{S4}$$

where $\mathrm{Tr_a}[\cdots]$ denotes the trace operation over atomic states, ρ^{F} is the field density matrix and ρ^{AF} is the atom-field density matrix. The exchange terms between atoms in $e^{-\frac{1}{2}(g\tau)^2\sum_{i=1}^{N-1}\sum_{j=i+1}^{N}\left(\sigma_i^\dagger\sigma_j - \sigma_i\sigma_j^\dagger\right)}$ appear quite complicated, but they only affect the state of atoms, which will be traced out and thus do not affect the field state. Therefore, Eq. (S4) can be simplified as

$$\rho^{\mathrm{F}}(\tau) \simeq e^{\mathcal{O}((g\tau)^3)} \mathrm{Tr_a}\left[U_N\cdots U_2 U_1 \rho^{\mathrm{AF}}(0) U_1^\dagger U_2^\dagger \cdots U_N^\dagger\right], \tag{S5}$$

where the N-atom evolution operator can be simplified as sequentially operating one-atom evolution operator for N times up to the second order of $g\tau$.

* kwan@phya.snu.ac.kr

The unitary time-evolution operator $U_k(\tau)$ associated with the kth atom is given by

$$\begin{aligned} U_k(\tau) &= e^{-iH_k\tau/\hbar} \\ &= \begin{pmatrix} \cos(\frac{1}{2}\Omega_n\tau) - (i\frac{\Delta}{\Omega_n})\sin(\frac{1}{2}\Omega_n\tau) & -(\frac{2ig}{\Omega_n})\sin(\frac{1}{2}\Omega_n\tau)a \\ -a^\dagger(\frac{2ig}{\Omega_{n-1}})\sin(\frac{1}{2}\Omega_{n-1}\tau) & \cos(\Omega_{n-1}\tau/2) - (\frac{i\Delta}{\Omega_{n-1}})\sin(\frac{1}{2}\Omega_{n-1}\tau) \end{pmatrix}, \end{aligned} \tag{S6}$$

where $\Omega_n \equiv \sqrt{4g^2(n+1) - \Delta^2}$. In the experiment, the cavity was resonant with the atom, so we let $\Delta = 0$ from now on. By expanding the sine and cosine terms in $g\tau$ in Eq. (S6) under the condition of $\sqrt{\langle n\rangle}g\tau \ll 1$ and assuming that the internal states of all atoms are identical with common $\rho_{\rm ee}, \rho_{\rm gg}$ and $\rho_{\rm eg}$, we can evaluate Eq. (S5) as

$$\begin{aligned} \rho^{\rm F}(\tau) = \rho^{\rm F}(0) & \\ & - \frac{1}{2}N(g\tau)^2\rho_{\rm ee}\left(aa^\dagger\rho^{\rm F}(0) + \rho^{\rm F}(0)aa^\dagger - 2a^\dagger\rho^{\rm F}(0)a\right) \\ & - \frac{1}{2}N(g\tau)^2\rho_{\rm gg}\left(a^\dagger a\rho^{\rm F}(0) + \rho^{\rm F}(0)a^\dagger a - 2a\rho^{\rm F}(0)a^\dagger\right) \\ & + iN(g\tau)\rho_{\rm eg}[\rho^{\rm F}(0), a^\dagger] + iN(g\tau)\rho_{\rm ge}[\rho^{\rm F}(0), a] + \mathcal{O}\left((g\tau)^3\right). \end{aligned} \tag{S7}$$

In the case of a single atom, the field density matrix after an interaction time τ is given by

$$\rho^{\rm F}_{N=1}(\tau) = {\rm Tr}_{\rm a}[U_1(\tau)\rho^{\rm AF}_{N=1}(0)U_1^\dagger(\tau)], \tag{S8}$$

and its series expansion in $g\tau$ results in nothing but the expression in Eq. (S7) with substitution $N = 1$.

If τ is shorter than the characteristic time of field growth or decay, the time derivative of density matrix can be approximated as $\dot{\rho}^{\rm F}(t) \simeq \frac{\rho^{\rm F}(\tau)-\rho^{\rm F}(0)}{\tau}$, yielding

$$\begin{aligned} \dot{\rho}^{\rm F}(t) = \gamma_{\rm inj}(g\tau)^2\rho_{\rm ee}\mathcal{L}[a^\dagger]\rho^{\rm F}(0) + \gamma_{\rm inj}(g\tau)^2\rho_{\rm gg}\mathcal{L}[a]\rho^{\rm F}(0) & \\ + i\gamma_{\rm inj}(g\tau)\rho_{\rm eg}[\rho^{\rm F}(0), a^\dagger] + i\gamma_{\rm inj}(g\tau)\rho_{\rm ge}[\rho^{\rm F}(0), a] & \\ + 2\gamma_{\rm c}\mathcal{L}[a]\rho^{\rm F}(0) & \end{aligned} \tag{S9}$$

where $\gamma_{\rm inj} = N/\tau$ is the atomic injection rate for multiple atoms. The Lindblad operator is defined as $\mathcal{L}[O]\rho = O\rho O^\dagger - \frac{1}{2}\left(O^\dagger O\rho + \rho O^\dagger O\right)$ for an arbitrary operator O. We have also included the cavity damping by adding $2\gamma_{\rm c}\mathcal{L}[a]\rho^{\rm F}(0)$ term in Eq. (S9), where $2\gamma_{\rm c}$ is the cavity decay rate (of intensity). We now define a modified decay rate $\Gamma'_{\rm c} \equiv 2\gamma_{\rm c} + \gamma_{\rm inj}(\rho_{\rm gg} - \rho_{\rm ee})(g\tau)^2 = 2\gamma_{\rm c}[1 + (1/2 - \rho_{\rm ee})(g\tau)^2N_{\rm c}]$ with $N_{\rm c} \equiv \gamma_{\rm inj}/\gamma_{\rm c}$ and a thermal photon number $n_{\rm th} \equiv \gamma_{\rm inj}(g\tau)^2\rho_{\rm ee}/\Gamma'_{\rm c} = (\gamma_{\rm c}/\Gamma'_{\rm c})\rho_{\rm ee}(g\tau)^2N_{\rm c}$. Equation (S9) can then be written as

$$\dot{\rho}^{\rm F}(t) = \Gamma'_{\rm c}n_{\rm th}\mathcal{L}[a^\dagger]\rho^{\rm F} + \Gamma'_{\rm c}(n_{\rm th}+1)\mathcal{L}[a]\rho^{\rm F} + i\gamma_{\rm inj}(g\tau)[\rho^{\rm F}, \rho_{\rm eg}a^\dagger + \rho_{\rm ge}a] \tag{S10}$$

We are interested in the steady state solution $\rho^{\rm F}_{\rm ss}$, which is obtained by letting $\dot{\rho}^{\rm F}(t) = 0$. When $\rho_{\rm eg} = 0$, we have

$$\Gamma'_{\rm c}n_{\rm th}\mathcal{L}[a^\dagger]\rho^{\rm F}_{\rm ss} + \Gamma'_{\rm c}(n_{\rm th}+1)\mathcal{L}[a]\rho^{\rm F}_{\rm ss} = 0, \tag{S11}$$

and the solution is

$$\rho^{\rm F}_{\rm ss} = \sum_k \frac{n^k_{\rm th}}{(n_{\rm th}+1)^{k+1}}\left|k\right\rangle\left\langle k\right| \equiv \rho^{\rm F}_{\rm th} \tag{S12}$$

and the resulting mean photon number is $\langle n\rangle = {\rm Tr}[a^\dagger a\rho^{\rm F}_{\rm ss}] = n_{\rm th}$, indicating the thermal photon number $n_{\rm th}$ is nothing but the mean photon number due to the population pumping only. In the presence of the polarization pumping, *i.e.*, $\rho_{\rm eg} \neq 0$, we have

$$\Gamma'_{\rm c}n_{\rm th}\mathcal{L}[a^\dagger]\rho^{\rm F}_{\rm ss} + \Gamma'_{\rm c}(n_{\rm th}+1)\mathcal{L}[a]\rho^{\rm F}_{\rm ss} + i\gamma_{\rm inj}(g\tau)[\rho^{\rm F}_{\rm ss}, \rho_{\rm eg}a^\dagger + \rho_{\rm ge}a] = 0. \tag{S13}$$

In this case, we introduce a thermal coherent state $\rho^{\rm F}_{\rm ss} = D(\alpha)\rho^{\rm F}_{\rm th}D^\dagger(\alpha)$ as an ansatz for the steady-state solution. The displacement operator is defined as $D(\alpha) \equiv e^{\alpha a^\dagger - \alpha^* a}$ with the parameter α is expected to be proportional to $\rho_{\rm eg}$, and thus if $\rho_{\rm eg} = 0$, we recover $\rho^{\rm F}_{\rm ss} = \rho^{\rm F}_{\rm th}$. If Eq. (S13) can be satisfied by a unique complex number α, then our ansatz is justified.

In order to find α satisfying Eq. (S13), let us apply $D^\dagger(\alpha)$ on the left and $D(\alpha)$ on the right side of Eq.(S13). Using the relation $D^\dagger(\alpha)aD(\alpha) = a+\alpha$ and $D^\dagger(\alpha)a^\dagger D(\alpha) = a^\dagger + \alpha^*$, we can show

$$\begin{aligned} D^\dagger(\alpha)\mathcal{L}[a]\rho_{\rm ss}^{\rm F}D(\alpha) &= \mathcal{L}[a]\rho_{\rm th}^{\rm F} + \tfrac{1}{2}[\rho_{\rm th}^{\rm F}, \alpha a^\dagger - \alpha^* a] \\ D^\dagger(\alpha)\mathcal{L}[a^\dagger]\rho_{\rm ss}^{\rm F}D(\alpha) &= \mathcal{L}[a^\dagger]\rho_{\rm th}^{\rm F} - \tfrac{1}{2}[\rho_{\rm th}^{\rm F}, \alpha a^\dagger - \alpha^* a], \end{aligned} \tag{S14}$$

and thus we obtain

$$\begin{aligned} 0 &= \Gamma_{\rm c}' n_{\rm th}\mathcal{L}[a^\dagger]\rho_{\rm th}^{\rm F} + \Gamma_{\rm c}'(n_{\rm th}+1)\mathcal{L}[a]\rho_{\rm th}^{\rm F} + \tfrac{1}{2}\Gamma_{\rm c}'[\rho_{\rm th}^{\rm F}, \alpha a^\dagger - \alpha^* a] \\ &\quad + i\gamma_{\rm inj}(g\tau)[\rho_{\rm th}^{\rm F}, \rho_{\rm eg}(a^\dagger + \alpha^*) + \rho_{\rm ge}(a+\alpha)] \\ &= \tfrac{1}{2}\Gamma_{\rm c}'[\rho_{\rm th}^{\rm F}, \alpha a^\dagger - \alpha^* a] + i\gamma_{\rm inj}(g\tau)[\rho_{\rm th}^{\rm F}, \rho_{\rm eg}a^\dagger + \rho_{\rm ge}a] \\ &= \left[\rho_{\rm th}^{\rm F}, \left(i\gamma_{\rm inj}g\tau\rho_{\rm eg} + \tfrac{1}{2}\Gamma_{\rm c}'\alpha\right)a^\dagger + \left(i\gamma_{\rm inj}g\tau\rho_{\rm ge} - \tfrac{1}{2}\Gamma_{\rm c}'\alpha^*\right)a\right], \end{aligned} \tag{S15}$$

where in obtaining the second equality we used the result in Eq. (S11) with $\rho_{\rm ss}^{\rm F} = \rho_{\rm th}^{\rm F}$. Equation (S15) is satisfied if and only if

$$\alpha = -\frac{2i\gamma_{\rm inj}(g\tau)\rho_{\rm eg}}{\Gamma_{\rm c}'} = -i\frac{2\gamma}{\Gamma_{\rm c}'}\rho_{\rm eq}(g\tau)N_{\rm c}. \tag{S16}$$

The mean photon number is given by $\langle n\rangle = {\rm Tr}[a^\dagger a\rho_{\rm ss}^{\rm F}]$, which is evaluated as

$$\begin{aligned} \langle n\rangle &= {\rm Tr}[a^\dagger a D(\alpha)\rho_{\rm th}^{\rm F}D^\dagger(\alpha)] = {\rm Tr}[D^\dagger(\alpha)a^\dagger D(\alpha)D^\dagger(\alpha)aD(\alpha)\rho_{\rm th}^{\rm F}] \\ &= {\rm Tr}[(a^\dagger+\alpha^*)(a+\alpha)\rho_{\rm th}^{\rm F}] = {\rm Tr}[a^\dagger a\rho_{\rm th}^{\rm F} + |\alpha|^2\rho_{\rm th}^{\rm F}] \\ &= n_{\rm th} + |\alpha|^2 = n_{\rm th} + n_{\rm sr} \end{aligned} \tag{S17}$$

proving Eq. (2).

The photon number distribution of the field is given by

$$P_n = \langle n|D(\alpha)\rho_{\rm th}^{\rm F}D^\dagger(\alpha)|n\rangle = \frac{1}{\pi n_{\rm th}}\int d^2\alpha'\; e^{-|\alpha'|^2/n_{\rm th}}e^{-|\alpha'+\alpha|^2}\frac{|\alpha'+\alpha|^{2n}}{n!}. \tag{S18}$$

In the integral, the first exponential factor $e^{-|\alpha'|^2/n_{\rm th}}$ is appreciable in a range of $|\alpha'|^2 < n_{\rm th}$. If $|\alpha|^2 \gg n_{\rm th}$, the rest of the integrand is a slowly varying function of α' where as the first exponential factor is sharply peaked at $\alpha' = 0$ like a delta function. Therefore, we can evaluate the rest of the integrand at $\alpha' = 0$ and take it out of the integral, and the result is

$$P_n \simeq \left[e^{-|\alpha|^2}\frac{|\alpha|^{2n}}{n!}\right]\frac{1}{\pi n_{\rm th}}\int d^2\alpha'\; e^{-|\alpha'|^2/n_{\rm th}} = e^{-|\alpha|^2}\frac{|\alpha|^{2n}}{n!}, \tag{S19}$$

which is the Poisson distribution with a mean photon number of $|\alpha|^2 = n_{\rm sr}$.

How close the actual distribution is to that of Poisson can be characterized by the second-order correlation function at zero delay time, which is given by

$$\begin{aligned} g^{(2)}(0) &= \frac{\langle a^\dagger a^\dagger aa\rangle}{\langle a^\dagger a\rangle^2} = \frac{{\rm Tr}[a^\dagger a^\dagger aa\rho_{\rm ss}^{\rm F}]}{{\rm Tr}[a^\dagger a\rho_{\rm ss}^{\rm F}]^2} = \frac{{\rm Tr}[a^\dagger a^\dagger aaD(\alpha)\rho_{\rm th}^{\rm F}D^\dagger(\alpha)]}{(n_{\rm th}+|\alpha|^2)^2} \\ &= \frac{{\rm Tr}[D^\dagger(\alpha)a^\dagger D(\alpha)D^\dagger(\alpha)a^\dagger D(\alpha)D^\dagger(\alpha)aD(\alpha)D^\dagger(\alpha)aD(\alpha)\rho_{\rm th}^{\rm F}]}{(n_{\rm th}+|\alpha|^2)^2} \\ &= \frac{{\rm Tr}[(a^\dagger+\alpha)^2(a+\alpha^*)^2\rho_{\rm th}^{\rm F}]}{(n_{\rm th}+|\alpha|^2)^2} = \frac{{\rm Tr}[a^{\dagger 2}a^2\rho_{\rm th}^{\rm F}] + 4|\alpha|^2{\rm Tr}[a^\dagger a\rho_{\rm th}^{\rm F}] + |\alpha|^4}{(n_{\rm th}+|\alpha|^2)^2} \\ &= \frac{2n_{\rm th}^2 + 4|\alpha|^2 n_{\rm th} + |\alpha|^4}{(n_{\rm th}+|\alpha|^2)^2} = 1 + \frac{1+2M}{(1+M)^2}, \end{aligned} \tag{S20}$$

where $M = n_{\rm sr}/n_{\rm th} = |\alpha|^2/n_{\rm th}$ is a measure of the dominance of the superradiance over thermal radiation. For $M \gg 1$, $g^{(2)}(0) \simeq 1$, resulting in the Poisson distribution. After the modified decay time $1/\Gamma_{\rm c}'$, $g^{(2)}(t)$ would converge to unity. Therefore,

$$g^{(2)}(t) = 1 + \frac{1+2M}{(1+M)^2}e^{-\Gamma_{\rm c}' t}. \tag{S21}$$
